# Comment on: ‘Triceps skinfold‐albumin index significantly predicts the prognosis of cancer cachexia: A multicentre cohort study’ by Yin et al.

**DOI:** 10.1002/jcsm.13304

**Published:** 2023-08-07

**Authors:** Ping'an Ding, Haotian Wu, Jiaxiang Wu, Chenyu Sun, Muzi Meng, Peigang Yang, Yang Liu, Lingjiao Meng, Qun Zhao

**Affiliations:** ^1^ The Third Department of Surgery The Fourth Hospital of Hebei Medical University Shijiazhuang China; ^2^ Hebei Key Laboratory of Precision Diagnosis and Comprehensive Treatment of Gastric Cancer Shijiazhuang China; ^3^ Department of Thyroid and Breast Surgery The Second Affiliated Hospital of Anhui Medical University Hefei China; ^4^ UK Program Site American University of the Caribbean School of Medicine Preston UK; ^5^ Bronxcare Health System The Bronx NY USA; ^6^ Research Center of the Fourth Hospital of Hebei Medical University Shijiazhuang China

The study by Yin et al.^1^ is greatly appreciated for their contributions to research, particularly in developing the triceps skinfold‐albumin index (TA). This new comprehensive index combines fat mass and nutritional status to evaluate malnutrition and has been identified as independently associated with the prognosis of patients with cancer cachexia. Yin et al.^1^ successfully identified different cut‐off values of TA for each gender, which divided patients into normal and low groups. The different groups showed significant differences in prognostic effects, with normal TA patients being significantly associated with lower mortality and the opposite association found for lower TA patients. Additionally, TA demonstrated a wide discrimination performance for the prognosis of patients of all ages. This study is particularly meaningful for two main reasons. Firstly, despite being calculated by only two sample parameters, TA's accuracy for predicting the prognosis of patients with cancer cachexia is higher than that of previous predictive indices, such as NRI, PNI, and SII. Furthermore, TA is cost‐effective and easy to use. Secondly, the gender‐specific cut‐off values of TA are consistent with the differences in nutrition between men and women. Moreover, this prospective, large sample, and geographically multi‐centre cohort study ensures reliable confirmation of the results. Therefore, based on these aspects, TA may be considered a clinically meaningful and promising indicator for predicting the prognosis of patients with cancer cachexia.

Nonetheless, the generalizability of the previous findings of TA was not confirmed in patients with diverse cancer types or undergoing various treatments. To address this gap, we investigated the clinical applicability of TA as a predictive tool for cancer cachexia in patients with locally advanced gastric cancer (LAGC) across multiple prospective cohorts (NCT01516944, NCT02555358, NCT03349866, and NCT01962246) registered in our institution. The study included 1266 LAGC patients, of which 898 (70.93%) had complete serum albumin values and detailed triceps skinfold thickness (mm) data. Of these patients, 188 (20.94%) were diagnosed with cancer cachexia based on the 2011 International Consensus on Cancer Cachexia criteria outlined by Fearon et al.^2^ The median age of the patients diagnosed with cancerous cachexia was 60 years (interquartile range [IQR], 35–77), with 125 (66.49%) males and 63 (33.51%) females. Our analysis revealed that mean TA was lower in males than in females in the cohort (51.8 vs. 56.3). We stratified the patients into high and low TA groups based on the optimal cut‐off values (male: TA < 45.6, female: TA < 49.9) established in the previous study by Yin et al.^1^ Out of 188 patients, 57 (30.32%) were classified in the low TA group. During a median follow‐up of 65.8 months (12.9–109.7 months), 20 (36.36%) patients with postoperative pathological stage II and 64 (48.12%) of stage III died. Our results showed that patients in the high TA group had significantly higher overall survival (OS) rates than those in the low TA group (61.83% vs. 40.35%, *P* = 0.004) (Figure 1A). Additionally, patients in the high TA group had superior disease‐free survival (DFS) rates compared with those in the low TA group (56.49% vs. 28.07%, *P* < 0.001) (Figure 1D). Subgroup analysis based on tumour pTNM staging also demonstrated that high TA was associated with prolonged OS and DFS in both stage II and III patients compared with those in the low TA group [(stage II: OS: 70.59% vs. 52.38%, *P* = 0.044; DFS: 64.71% vs. 47.62%, *P* = 0.027); (stage III: OS: 58.76% vs. 33.33%, *P* = 0.002; DFS: 53.61% vs. 16.67%, *P* < 0.001)] (Figure 1B,C,E,F). Our findings suggest that TA can be a useful tool to predict the prognosis and recurrence status of LAGC patients with cancer cachexia, similar to the original study by Yin et al.^1^

**Figure 1:**
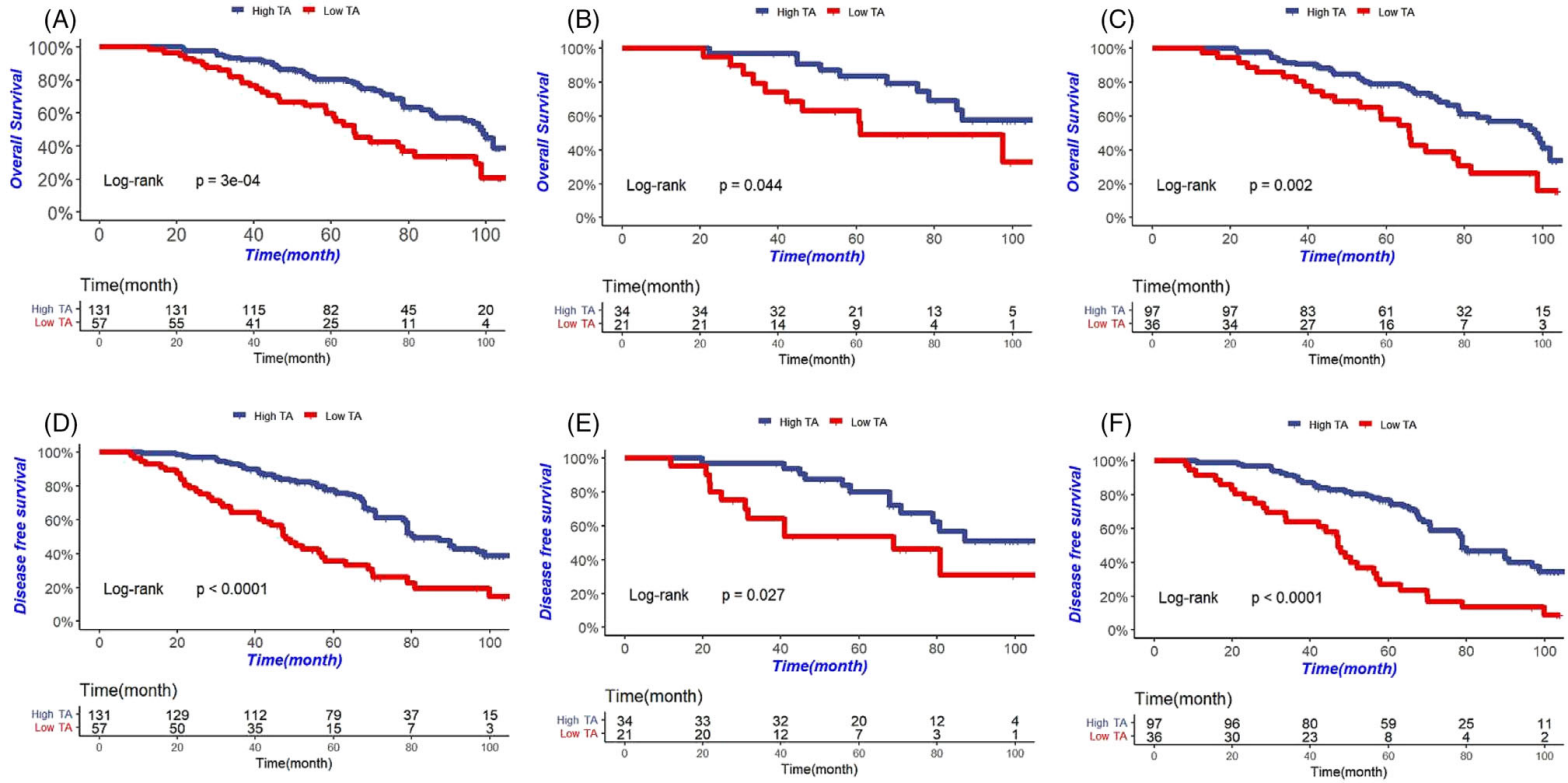
Relationship between triceps skinfold‐albumin index (TA) and survival of patients with cancer cachexia in LAGC. (A) Kaplan–Meier curves for overall survival in the cohort. (B) Kaplan–Meier curves for overall survival of patients in the cohort with stage II. (C) Kaplan–Meier curves for overall survival of patients in the cohort with stage III. (D) Kaplan–Meier curves for disease‐free survival in the cohort. (E) Kaplan–Meier curves for disease‐free survival of patients in the cohort with stage II. (F) Kaplan–Meier curves for disease‐free survival of patients in the cohort with stage III.

Patients with cancer often suffer from concurrent cancer cachexia, which can have a negative impact on their prognosis and survival time.^3^, ^4^ Therefore, it is crucial to identify reliable and cost‐effective prognostic markers for patients with cancer cachexia. Although protein and inflammatory biomarkers have been studied to predict the prognosis of LAGC patients with cachexia,^5^, ^6^ their high cost and single influencing aspect limit their clinical application. TA, as a comprehensive prognostic predictor that combines fat mass and nutritional status of cancer cachexia, offers a meaningful prognostic stratification and has been demonstrated to be clinically significant for LAGC patients with cachexia of different tumour pathological or different clinical therapies.

We would like to express our gratitude to Liangyu Yin et al. for their valuable contribution to improving the prognostication of cancer cachexia and for providing a clinically reliable biomarker. Their research has demonstrated that TA is a comprehensive marker that can reflect the fat mass, nutritional status, and inflammatory status of LAGC patients with cachexia. This finding may help clinicians in formulating effective treatment strategies and improving the clinical management of LAGC patients with cachexia. In our own study, we have found that TA is a useful predictor of prognosis for LAGC patients with cachexia. By using TA, we can provide more precise prognostic guidance and improve the survival time of our patients.

## Funding

This work was supported by the Cultivating Outstanding Talents Project of Hebei Provincial Government Fund (No. 2019012) and Hebei Public Health Committee County‐Level Public Hospitals Suitable Health Technology Promotion and Storage Project (No. 2019024).
